# Targeting of Notch, IL-1, and leptin has therapeutic potential in xenograft colorectal cancer

**DOI:** 10.55730/1300-0152.2663

**Published:** 2023-08-10

**Authors:** Rumeysa ÖZYURT, Nilüfer ERKASAP, Mete ÖZKURT, Serdar ERKASAP, Konstantinos DİMAS, Ayşe ÇAKIR GÜNDOĞDU, Engin ULUKAYA

**Affiliations:** 1 Department of Experimental Therapeutics, MD Anderson Cancer Center, Houston, TX, USA; 2 Department of Physiology, Eskişehir Osmangazi University Medical Faculty, Eskişehir, Turkiye; 3 Department of General Surgery, Eskişehir Osmangazi University Medical Faculty, Eskişehir, Turkiye; 4 Department of Pharmacology, School of Health Science, Thessaly University, Larissa, Greece; 5 Department of Histology and Embrology, Kütahya Health Sciences University Medical Faculty, Kütahya, Turkiye; 6 Department of Clinical Biochemistry, Faculty of Medicine, İstinye University, İstanbul, Turkiye

**Keywords:** Colorectal cancer, Notch, IL-1, leptin, proinflammatory, proangiogenic

## Abstract

**Background/aim:**

Colorectal cancer (CRC) is a fatal malignancy type and its occurence still needs to be explored mechanistically. Notch, IL-1, and leptin crosstalk is reported to play a role in the proliferation, migration, and expression of proangiogenic molecules. In this study, we aimed to investigate the effect of inhibition of Notch, IL-1, and leptin on CRC.

**Materials and methods:**

To generate colorectal cancer tumor xenografts, 1 × 10^7^ cells from exponentially growing cultures of HCT-15 cells were injected subcutaneously, at the axillary region of the left and right rear flanks of forty NOD.CB17-Prkdc^scid^/J (NOD/SCID) female mice. The mice were then monitored for the development of tumors and were randomly divided into five groups when tumor sizes reached a volume of approximately 150 mm^3^. Mice were used to determine the effectiveness of the gamma-secretase inhibitor (DAPT, Notch inhibitor), the interleukin-1 receptor antagonist (Anakinra) and the leptin receptor antagonist (Allo aca) against tumor growth. The mice were euthanized by CO_2_ inhalation immediately after the treatments finished, and all efforts were made to minimize suffering. Tumors were excissed for RT-PCR and histological analysis.

**Results:**

There is an intact Notch, IL-1, and leptin signaling axis, and in vivo antagonism of Notch, IL-1, and leptin affects mRNA and protein expression of inflammatory and angiogenic molecules.

**Conclusion:**

Present data suggest that targeting Notch, IL-1, and leptin may be possesses therapeutic potential in CRC.

## 1.Introduction

Colorectal cancer (CRC) is the third most commonly-diagnosed cancer among men and women worldwide(Siegel et al., 2023). There are many risk factors for CRCdevelopment, one of which is changes in the adipokinemetabolism. Studies have showed both mechanistic andclinical roles for adipokines in tumorigenic signalingpathways directly or via intermediate molecules (Tewariet al., 2022). Affected signaling pathways regulate theexpression of oncogene, or tumor suppressor genes, thatregulate the cell cycle (Pham and Park, 2021). Leptin,a product of the obesity (ob) gene, is one of the mostspecific adipokines released from white adipose tissue,which normally functions as an energy sensor (Songet al.,2018). In addition, leptin acts like mitogens by inhibitingapoptosis and increasing tumor development (Endo et al.,2011). Changes in blood leptin levels have been observed in most types of cancer, including CRC (Tong et al., 2008; N.Erkasap et al., 2013).

Notch signaling is a hallmark of the cancer, which isessential for normal continuity and homeostasis of theintestinal epithelium (Dunkin et al., 2018). As activatingmutations in Notch receptors are unusual in colorectalcancer, increasing evidence highlights the importanceof Notch signaling in intestinal tumorigenesis (Dunkinet al., 2018). JAG1/Notch1 signaling controls oncogenicprocesses in different cell types and cellular contexts.Recent studies revealed the involvement of Jagged1, aNotch ligand, in CRC development (Rodilla et al., 2009;Dai et al., 2014). These data provide a promising approachfor inhibition of the Notch pathway for the treatment ofCRC (Guilmeau, 2012). One approach that is currentlyuseful in clinical trials is to inhibit the degradation of Notch receptors with γ-secretase inhibitors. These agents have demonstrated therapeutic benefit for CRC (Akiyoshi et al., 2008). However, the cause of Notch pathway dysregulation in intestinal tumorigenesis remains unclear.

Recent data revealed that leptin induces expression of Notch family components associated with IL-1 signaling in breast cancer (Guo and Gonzalez-Perez, 2011; Battle et al., 2014). Leptin also induces the expression of Notch receptors and ligands in pancreatic and endometrial cancer (Daley-Brown et al., 2016; Harbuzariu et al., 2017). In addition, leptin and IL-1 induced signals have been reported to be interrelated in many pathological conditions, such as tumor inflammation, proliferation, and angiogenesis (Newman and Gonzalez-Perez, 2014). The synergistic action between leptin and IL-1 can increase VEGF expression, an angiogenic molecule in breast cancer (Gonzalez-Perez et al., 2010). A novel signal crosstalk between leptin, Notch, and IL-1 (Notch, IL-1, and leptin crosstalk, NILCO) has been demonstrated to drive leptin-induced oncogenic effects in breast cancer. NILCO has been shown to play a role in the proliferation, migration, and expression of proangiogenic molecules in cancers, such as breast cancer, and endometrial and pancreatic cancers (Guo and Gonzalez-Perez, 2011; Harbuzariu et al., 2017; Daley-Brown et al., 2019). In addition, it has been suggested that VEGF/VEGFR2 gene expression, which plays a role in NILCO mediated angiogenesis, may contribute significantly to tumor development (Guo and Gonzalez-Perez, 2011). Our previus study suggests that Notch, IL-1, and leptin may serve a crucial role in the development of colorectal cancer (Erkasap et al., 2021). Here, we aimed to elucidate the effects of Notch, IL-1, and leptin inhibition on CRC. Present data suggest that crosstalk between leptin, IL-1, and Notch may be critical mediators in the generation of proinflammatory and proangiogenic signals and its inhibition possesses therapeutic potential in CRC.

## 2. Materials and methods

### 2.1. Cell culture conditions

An HCT-15 colorectal adenocarcinoma cell line was obtained from ATCC (Manassas, VA, USA). Cancer cells were adapted to proliferate in RPMI-1640 medium, supplemented with 5% heat-inactivated fetal calf serum, 2 mM L-glutamine and antibiotics. The cultures were grown at 37 °C in a humidified incubator with a 5% CO_2_ atmosphere and 95% humidity. The cells were subcultured at seventy-two h intervals using 0.25% trypsin/EDTA and were seeded in fresh media. The cells were regularly evaluated for Mycoplasma contamination, and all experiments were carried out with cells at 60%–80% confluence in culture flask.

### 2.2. Antagonists

γ-secretase inhibitor DAPT (GSI-IX) was purchased from Selleckchem (Cat No. 208255-80-5, Houston, TX, USA), IL-1 receptor antagonist Anakinra was purchased from TOCRIS (Cat No. 185413-30-3, Minneapolis, MN, USA) and the leptin receptor antagonist, Allo aca, was purchased from Peptides international (Cat No. PCS-32627-PI, Louisville, Kentucky, USA). The DAPT was dissolved in 4% DMSO/Safflower Oil, Anakinra and the Allo aca were dissolved in 10% DMSO/0.9% NaCl. Before use, all of the drugs were freshly dissolved.

### 2.3. Ethics approval

Forty nude athymic mice (NOD.CB17-Prkdc^scid^/J) were obtained from the University of Thessaly, Faculty of Medicine, Department of Pharmacology, Larissa, Greece. This study was carried out in strict accordance with the recommendations in the Guide for the Care and Use of Laboratory Animals under Greek law, with the EU Animal Care and Use Committee (EU/2010/63). The protocol was approved by the Committee on the Ethics of Animal Experiments of the University of Thessaly (Protocol Number: 5542/228006). The animals in the study were euthanized by CO_2_ inhalation, and all efforts were made to minimize suffering.

### 2.4. CRC tumor xenograft model

To generate colorectal cancer tumor xenografts, 1 × 10^7^ cells from exponentially growing cultures of HCT-15 cells were injected subcutaneously, according to the British practice of bilateral implants, at the axillary region of the left and right rear flanks into forty 6–8 week old female NOD.CB17-Prkdc^scid^/J (NOD/SCID) mice (mean wieght, 20 g) from the Dr DIMAS animal facility laboratory (University of Thessaly, Faculty of Medicine, Department of Pharmacology, Larissa, Greece). During the experimentation, all of the animals were kept in the Animal Unit of the Department of Pharmacology under specific pathogen-free (SPF) conditions, a 12-h light/12-h dark regime, a temperature of 21 °C and a relative humidity of 50% and were allowed access to water and food ad libitum. Each group consisted of eight mice of matching age and weight. The mice were then monitored for the development of tumors. The mice were used to determine the effectiveness of DAPT, Anakinra and Allo aca against tumor growth. DAPT was dissolved in 4% DMSO/Safflower Oil, and Anakinra and Allo aca were dissolved in 10% DMSO/0.9% NaCl. The mice were randomly divided into five groups when the tumor sizes reached a volume of approximately 150 mm^3^ as follows: Carrier-1 group (n = 8) that received 300 μL 4% DMSO/Safflower Oil via subcutaneous (s.c.) injections twice a week for fifteen days; Carrier-2 group (n = 8) that received 300 μL 10% DMSO/0.9% NaCl via s.c. injections twice a week for fifteen days; the DAPT group (n = 8) that received s.c. injection (300 μL DAPT 10 mg/kg/day) twice a week for fifteen days; the Anakinra group (n = 8) that received s.c injection (300 μL of Anakinra 2 mg/kg/day) twice a week for fifteen days; and the Allo aca group n = 8) that received s.c. injection (300 μL Allo aca 3 mg/kg/day) twice a week for fifteen days. Tumor volumes were calculated using the formula V (mm^3^) = a × b^2^/2 (a = length and b = width of the tumor as measured with a Vernier’s caliper). Tumor measurements and animal weighing were performed twice a week. The animals were euthanized by CO_2_ inhalation immediately after the treatments finished, and all efforts were made to minimize suffering. The tumors were excissed for further molecular analysis.

### 2.5. RNA extraction and reverse transcription PCR

Total RNA was isolated from colorectal tumor tissues using a GeneJet RNA Purification Kit (Thermo Scientific, USA). The concentration and purity of the RNA were measured using a NanoDrop 1000 (Thermo Scientific, USA). Isolated RNA samples were converted to complementary DNA (cDNA) using a RevertAid First Strand cDNA Synthesis Kit (Thermo Scientific, USA) at 42 °C for sixty min and 70 °C for five min. cDNA samples were stored at −80 °C until analysis. NOTCH1, JAGGED1, LEPTIN, ObRb, IL-1α, IL-1β, IL-1R, VEGF-A, VEGFR1, and VEGFR2 gene expressions were measured using an SYBR Green qPCR Kit (Thermo Scientific, USA). β-actin was used as an internal control. Relative differences in expression were determined using the comparative threshold cycle (2^−ΔΔCt^) method.

### 2.6. Histology

Tissue samples were collected and rinsed with a phosphate buffer solution. After being fixed in 10% neutral buffered formaldehyde for seventy-two h, the tissues were embedded in paraffin. Sections of 4 μm thickness were obtained from paraffin blocks on slides using a microtome (Leica SM 2000, Germany). The sections were deparaffinized using xylene and rehydrated using a graded ethanol series. The rehydrated sections were then stained with hematoxylin and eosin (H&E), viewed under a computerized photo-light microscope (Leica DM4000B, Germany) and coded for blind examination. Assessment criteria included lymphocytic infiltration, necrosis, and apoptosis. Five different visual areas were chosen randomly per section from each animal group and their average was used in the analysis of each of the criteria in tumor sections. The degree of lymphocytic infiltration was graded as follows: 0 = no infiltration; 1 = minimal (1%–10% of surface area); 2 = mild (10%–20%); 3 = moderate (>20%–50%); and 4 = strong (≥50%) (Grigoriadis et al., 2018). Similarly, necrotic scores from 0 to 3 were given according to the areal percentages of necrosis: 0 = absence of necrosis in the observed area; 1 = mild (necrosis with nuclear pyknosis); 2 = moderate (<25%); and 3 = severe (>25%) (Liang et al., 2017). Apoptotic cells were counted against the total number of tumor cells and an apoptotic index was calculated as the number of apoptotic cells/number of total cells × 100 (Kim et al., 2001).

To carry out immune-histochemical analyses, the paraffin sections were deparaffinized and dehydrated. Heat-induced antigen retrieval was performed by Citrate Buffer (pH 6.0), endogenous peroxidase activity was blocked with 3% H_2_O_2_, and the epitopes were stabilized using a serum blocking solution. The sections were then incubated overnight at 4 °C with PBS containing primary antibodies against NOTCH1 (1: 200; abcam-ab52627), IL-1α (1: 200; Santa Cruz Biotechnology-sc9983), and IL-1β (1: 100; abcam-9722). Biotinylated secondary antibody and streptavidin peroxidase (LifeTech) were incubated with the tissues for ten min at room temperature. PBS was used to wash the slides between the steps. The binding sites of antibody were visualized with DAB (DAB Chromogen/Substrate Kit, ScyTek). The sections were counterstained with Harris’s Hematoxylin, evaluated under a photo-light microscope (DM4000B Image Analyze System, Leica, Germany) and a Leica DFC280 plus camera, and a LAS software programme at a magnification of ×400. ImageJ software (version 1.52; National Institutes of Health, MD, USA) was used to assess the staining intensity of antibodies in the tumour tissues. Average signal levels in five areas of each tissue were measured, and the background (counterstained samples treated with secondary antibodies) was subtracted.

### 2.7. Statistics

Statistical analysis was performed using GraphPad software. The data was analyzed by the Kruskal-Wallis test or one-way analysis of variance with a post hoc Tukey’s or Dunn’s test. Differences with p-values <0.05 were considered significant. Data is presented as the mean or median ± standard error of the mean or median (25%, 75%).

## 3. Results

### 3.1. In vivo antitumor efficacy of Notch, IL-1, and leptin inhibition

To characterize the in vivo antitumor activity of DAPT, Anakinra and Allo aca, the tumor volume and body weight of the HCT-15 tumor-bearing mice was monitored. Initially, we determined the dose and duration of treatment based on previous in vivo studies (Harnack et al., 2010; Otvos et al., 2011; Kalantari et al., 2013; Pilot et al., 2020; Philp et al., 2021). Based on this dose and duration information, we followed 15 days of treatment, twice a week, as described in the method, for CRC xenograft experiments.

**Figure 1:**
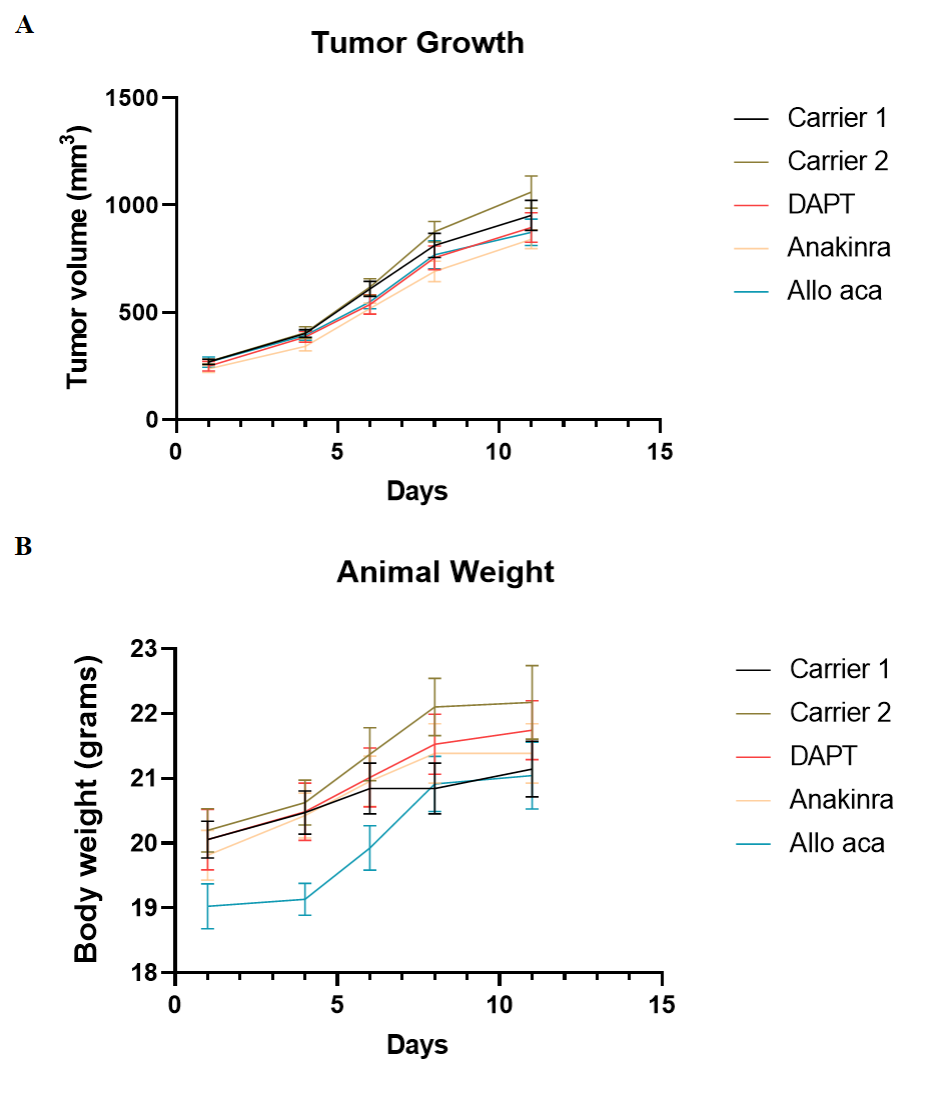
(A) The tumor volume and (B) body weight changes of Carrier 1, Carrier 2, DAPT, Anakinra and Allo aca treatments on the HCT-15 tumor-bearing mice. Data is shown as mean ± SE derived from a minimum of three independent experiments. ^*^p < 0.05, ^**^p < 0.01, and ***p < 0.001.

DAPT, Anakinra and Allo aca treatments had slightly inhibitory effect on tumour growth compared to controls (Figure 1A). Although Anakinra showed the most antigrowth effect among them, none of them were significant (p > 0.05). During the treatment period, mice in all groups gained weight, but no significant changes were observed (Figure 1A), (p > 0.05).

### 3.2. Inhibition of Notch, IL-1, and leptin affects mRNA and protein expression of inflammatory and angiogenic molecules

**Figure 2:**
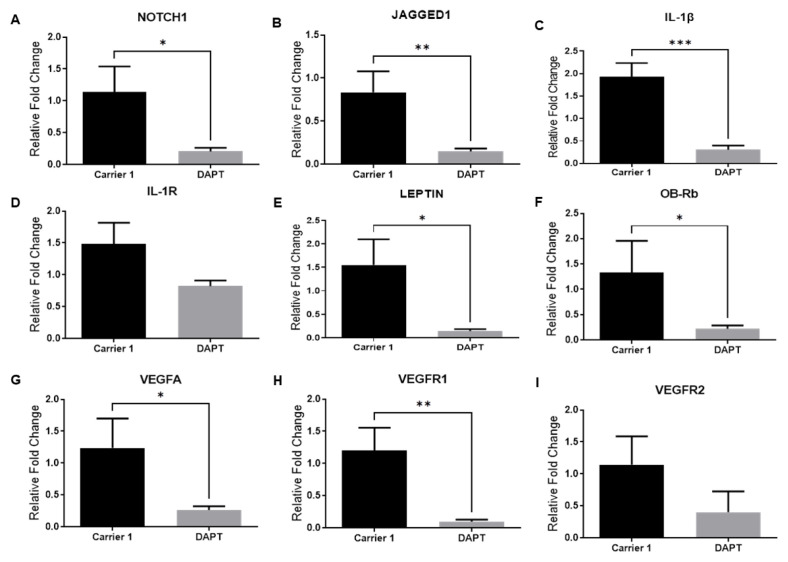
mRNA levels of NOTCH1 receptor, JAGGED1 ligand, IL-1β and IL-1R, LEPTIN, ObRb, VEGF-A, VEGFR1, and VEGFR2 (A-B-C-D-E-F-G-H-I respectively) in the γ-secretase inhibitor DAPT treatment group in CRC tumors. Levels of mRNA determined by real-time RT-PCR. β-actin was used as an internal control. Data is shown as mean ± SE derived from a minimum of three independent experiments. ^*^p < 0.05, ^**^p < 0.01, and ***p < 0.001.

**Figure 3:**
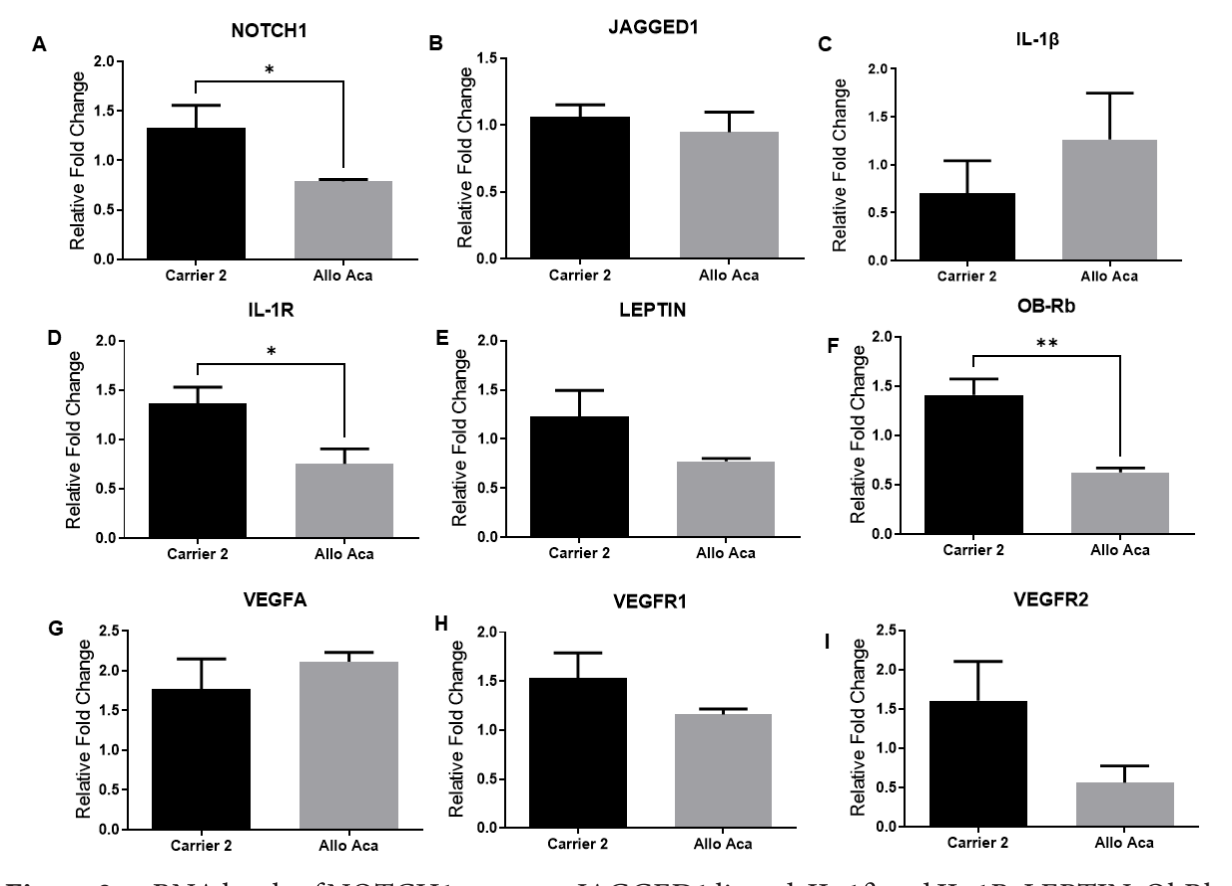
mRNA levels of NOTCH1 receptor, JAGGED1 ligand, IL-1β and IL-1R, LEPTIN, ObRb, VEGF-A, VEGFR1, and VEGFR2 (A-B-C-D-E-F-G-H-I respectively) in the LEPTIN receptor antagonist Allo aca treatment group in CRC tumors. Levels of mRNA were determined by real-time RT-PCR. β-actin was used as an internal control. Data is shown as mean ± SE derived from a minimum of three independent experiments: ^*^p < 0.05, ^**^p < 0.01, and ***p < 0.001.

**Figure 4:**
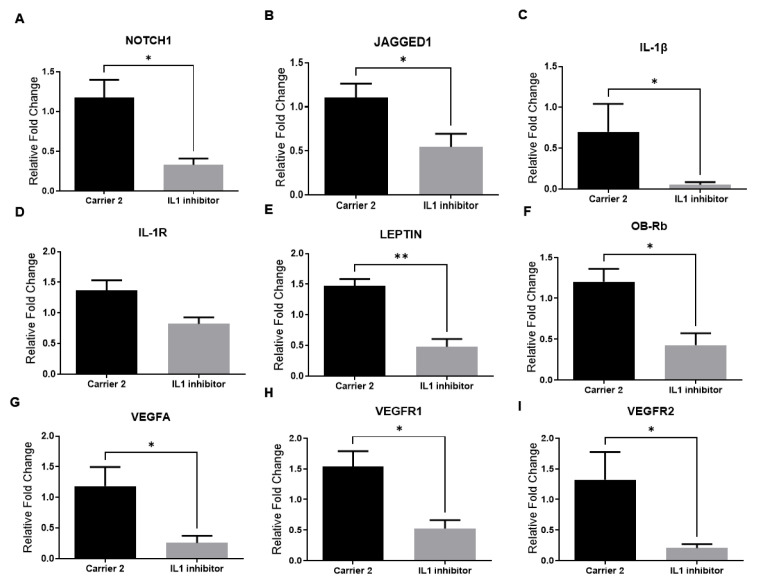
mRNA levels of NOTCH1 receptor, JAGGED1 ligand, IL-1β and IL-1R, LEPTIN, ObRb, VEGF-A, VEGFR1, and VEGFR2 (A-B-C-D-E-F-G-H-I respectively) in the IL1 receptor antagonist Anakinra treatment group in CRC tumors. Levels of mRNA were determined by real-time RT-PCR. β-actin was used as an internal control. Data is shown as mean ± SE derived from a minimum of three independent experiments: ^*^p < 0.05, ^**^p < 0.01, and ***p < 0.001.

First, we determined whether Notch, IL-1, and leptin signaling could be involved in the regulation of NOTCH1, JAGGED1, LEPTIN, ObRb, IL-1β, IL-1R, VEGF-A, VEGFR1, and VEGFR2 gene expression in colorectal tumor tissues. The blockade of Notch signaling by the γ-secretase inhibitor DAPT significantly reduced NOTCH1, JAGGED1, LEPTIN, ObRb, IL-1β, VEGF-A, and VEGFR1 mRNA levels in colorectal tumor tissues (Figure 2.). The inhibition of leptin signaling by the leptin receptor antagonist Allo aca reduced NOTCH1, IL-1R, and ObRb mRNA levels in colorectal tumor tissues. (Figure 3.). Furthermore, inhibition of IL-1 signaling by Anakinra decreased NOTCH1, JAGGED1, IL-1β, LEPTIN, ObRb, VEGFA, VEGFR1, and VEGFR2 expressions in colorectal tumors (Figure 4). These results suggest that a crosstalk between leptin, IL-1, and Notch in colorectal cancer.

### 3.3. IHC reveals the decreasing protein levels of NOTCH1, IL-1α, and IL-1β in the treatment groups

**Figure 5:**
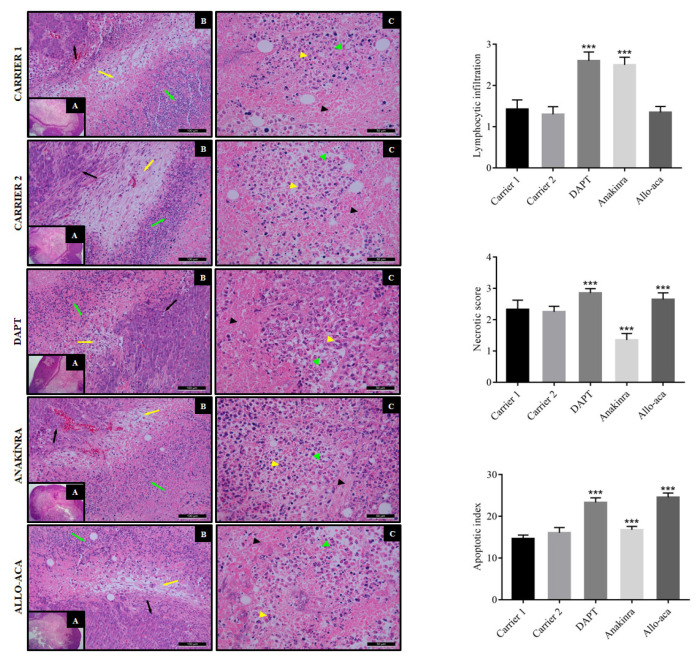
Representative photomicrographs of Hematoxylin and eosin-stained sections from the tumor tissues. Panoramic observation shows that tumor tissues consist of necrosis in the center, but more solid areas at the periphery (H&E, ×40): (A) Gland-like structures formed by atypical epithelial cells (black arrows), connective tissue (yellow arrows) and peritumoral lymphocytes (green arrows) (H&E, ×200); (B) Central necrotic areas (black arrowhead) surrounded by apoptotic tumor cells (yellow arrowhead) and intratumoral lymphocytes (green arrowhead) (H&E, ×400); (C) Effects of NILCO inhibitors on lymphocytic infiltration; (D) necrosis; (E) apoptosis; and (F) ^*^p < 0.05, ^**^p < 0.01, and ***p < 0.001.

H&E results reveal that tumors excised from CRC xenografts, developed by injecting HCT15 human colorectal cancer cells in NOD/SCID mice, are composed of a necrotic center with solid areas at the periphery (Figure 5A). In the solid areas, tubular gland-like structures form by atypical columnar epithelial cells, surrounded by well-vascularized cellular connective tissue, are observed. There is prominent peritumoral lymphocytic infiltration in all of the tissues (Figure 5B). Tumor cells with normal nuclear morphology, as well as apoptotic tumor cells with fragmented nucleus and intratumoral lymphocyte infiltration, are present around the necrotic areas in the center of the tumor tissues (Figure 5C).

Detailed examination of the H&E stained tumor sections show that inhibition of Notch signaling by DAPT and inhibition of IL1 signaling by Anakinra significantly increase lymphocytic infiltration in tumors (***p < 0.001 vs. carriers) (Figure 5D). However, no statistically significant difference in lymphocytic infiltration can be found between the leptin signaling Allo-aca-treated inhibitor and the carrier-treated mice. Tumor necrosis increases in the DAPT- and Allo-aca-treated tumors (***p < 0.001 vs. carrier), but it decreases significantly with Anakinra treatment (***p < 0.001 vs. carrier) (Figure 5E). In addition, a significant increase in apoptotic index is observed in all three treatment groups compared to the carriers (***p < 0.001) (Figure 5F).

**Figure 6:**
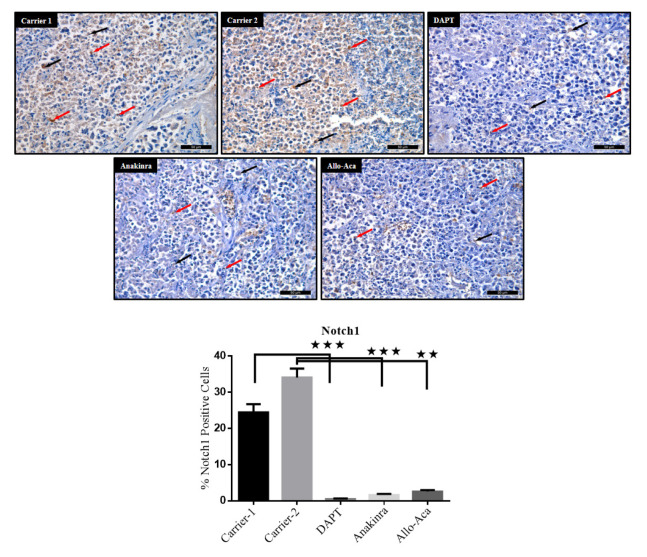
Immuno-histochemical analysis of NOTCH1 protein expression in tumor tissues. Strong cytoplasmic NOTCH1 immuno-reactivity (arrows) throughout the tumor cells in Carrier 1 and Carrier 2 receiving tumors, and weak cytoplasmic NOTCH1 immuno-reactivity in a small number of tumor cells in the DAPT, Anakinra and Allo aca treated tumors (DAB-Hematoxylin, ×400). ^*^p < 0.05, ^**^p < 0.01, and ***p < 0.001.

Subsequent and more detailed IHC analysis show that NOTCH1 is distributed in the cytoplasm and nuclei of tumor cells. The percentage of NOTCH1 immunoreactive cells is substantially higher in Carrier 1 and Carrier 2 groups, while NOTCH1 expression is significantly downregulated in the Allo aca, Anakinra and DAPT treated groups compared to the Carrier groups (p < 0.001). The percentage of NOTCH1 positive cells in the Allo aca and Anakinra treated mice is extremely similar, while it is significantly lower in the DAPT treated mice compared to these groups (Figure 6.).

**Figure 7:**
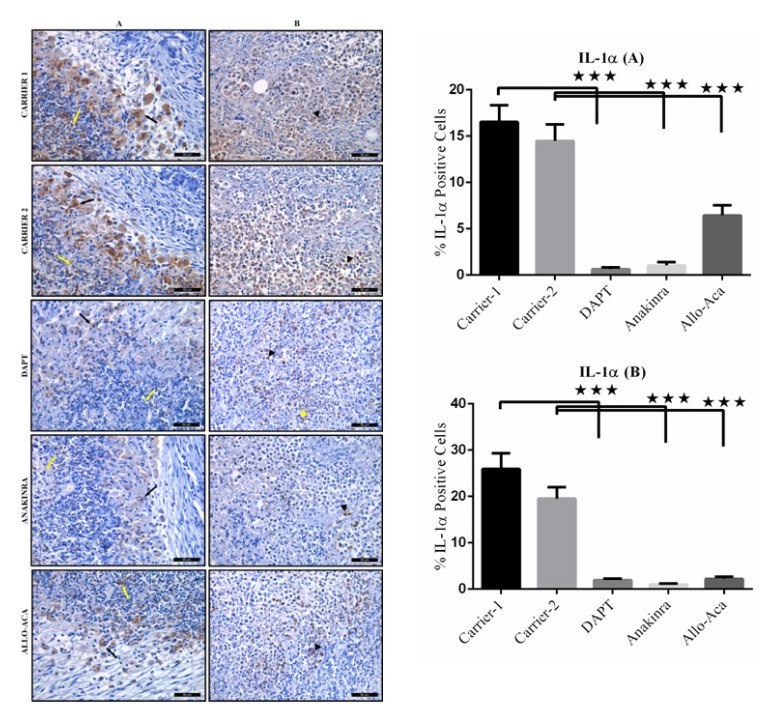
Immuno-histochemical analysis of IL-1α protein expression in tumor tissues. (A) Macrophages (black arrow) and lymphocytes (yellow arrow) show strong cytoplasmic IL-1α immune-reactivity in Carrier 1 and Carrier 2 receiving tumors, and weak immune-reaction in the DAPT, Anakinra and Allo aca treated tumors. (B) Strong cytoplasmic IL-1α immune-reactivity (arrowhead) in most tumor cells in Carrier 1 and Carrier 2 receiving tumors, and weak cytoplasmic IL-1α immunoreaction in a small number of tumor cells in the DAPT, Anakinra and Allo aca treated tumors (DAB-Hematoxylin, ×400). ^*^p < 0.05, ^**^p < 0.01, and ***p < 0.001.

**Figure 8:**
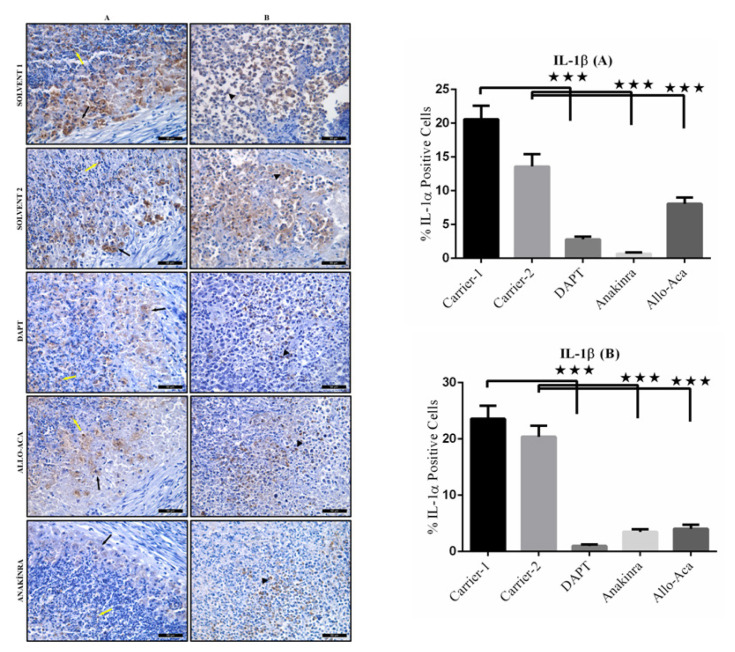
Immuno-histochemical analysis of IL-1β protein expression in tumor tissues. (A) Macrophages (black arrow) and lymphocytes (yellow arrow) show strong cytoplasmic IL-1β immuno-reaction in Carrier 1 and Carrier 2 receiving groups tumors and weak immuno-reaction in DAPT, Anakinra and Allo aca groups. (B) Strong cytoplasmic IL-1β immune-reaction (arrowhead) in most tumor cells in Carrier 1 and Carrier 2 receiving groups’ tumors and weak cytoplasmic IL-1β immune-reaction in a small number of tumor cells in the DAPT, Anakinra and Allo aca treated tumors (DAB-Hematoxylin, ×400). ^*^p < 0.05, ^**^p < 0.01, and ***p < 0.001.

IL-1α and IL-1β immune-reactivities are predominant in the cytoplasm of the macrophages, lymphocytes, and tumor cells. Representative examples of the microscopic immune-histochemical staining for determination of IL-1α and IL-1β expression qualitatively reveal a greater concentration of IL-1α and IL-1β in the Carrier groups. The tumors of Allo aca treated mice show similar IL-1α and IL-1β expression to that in the tumors of the DAPT treated mice. There is a marked reduction (p < 0.001) in the expression of IL-1α and IL-1β in the tumor tissues of Anakinra treated mice compared to all of the other groups (Figure 7 and Figure 8).

## 4. Discussion

Leptin, the most renowned adipokine, is one of the key risk factors for the several cancers, such as colorectal, esophageal, kidney, liver, pancreatic, thyroid, breast, and ovarian cancer (Al-Shibli et al., 2019; Cirillo et al., 2019; Sung et al., 2019). Leptin is an important proangiogenic, proinflammatory, and mitogenic marker with various functions that are not yet fully elucidated, and the effects of which are enhanced by cross-talk with cytokines/growth factors (Socol et al., 2022). Activation of ObR by leptin overexpression mediates inflammation, VEGF/VEGFR-2-dependent angiogenesis and, by so doing, cancer progression (Barone et al., 2016; Olea-Flores et al., 2018). In our previous study, we found that the serum leptin levels of patients with metastatic CRC were higher than those of nonmetastatic patients. (N. Erkasap et al., 2013). Currently, however, the insufficient data related to leptin’s relationship with the main pathogenic pathways of CRC indicate the necessity of further studies.

Leptin-ObR system is a potent activator of the IL-1 cytokine family in CRC, breast, endometrium and pancreatic cancer cells (Daley-Brown et al., 2017; Harbuzariu et al., 2018). In addition, leptin, and IL-1 crosstalk signals are interrelated in many pathological conditions such as tumor inflammation, proliferation, and angiogenesis (Newman and Gonzalez-Perez, 2014). The synergistic action between leptin and IL-1 can increase VEGF expression, an angiogenic molecule, in breast cancer (Gonzalez-Perez et al., 2010).

In current study, IL-1α and IL-1β expression are significantly downregulated in the Allo aca treated groups compared to the control groups. Meanwhile, we found that antagonism of leptin via Allo aca decreased ObRb, IL-1R, VEFR1 and VEGFR2 mRNA levels. Inhibition of IL1 signaling by Anakinra decreased IL-1β, LEPTIN, ObRb, VEGFA, VEGFR1, and VEGFR2 mRNA levels in CRC tumors. Furthermore, there is a marked reduction in the expression of IL-1α and IL-1β in the tumor tissues of Anakinra treated mice compared to control groups.

Previous studies have indicated that leptin up-regulates Notch signaling in breast and pancreatic cancer cells (Battle et al., 2014; Harbuzariu et al., 2017; Harbuzariu and Gonzalez-Perez, 2018). In particular, leptin-driven Notch and IL-1 signals mediate breast cancer cell proliferation, migration, invasion as well as chemoresistance (Guo and Gonzalez-Perez, 2011). We earlier reported that Notch, IL-1α, leptin, and VEGF/VEGFR-2 expressions were higher in human CRC and tyroid tumor tissue compared to normal tissue (Erkasap et al., 2020; Erkasap et al., 2021). Here, we found that NOTCH1 mRNA and protein expression is significantly downregulated in the Allo aca treated group compared to the control groups. In addition, we indicated that downregulation of IL1 signaling by Anakinra reduced NOTCH1 and JAGGED1 expressions in colorectal tumor tissues compared to control groups. Notch signaling is a hallmark of the cancer, which is essential for normal continuity and homeostasis of the intestinal epithelium (Dunkin et al., 2018). As activating mutations in Notch receptors are abnormal in colorectal cancer, increasing evidence underlines the value of Notch signaling in intestinal tumorigenesis (Dunkin et al., 2018; Jackstadt et al., 2019). In present study, inhibition of Notch signaling by the γ-secretase inhibitor DAPT significantly reduced NOTCH1, JAGGED1, LEPTIN, ObRb, IL-1β, VEGF-A, and VEGFR1 mRNA levels in colorectal tumor tissues compared to control groups. Morever, the percentage of NOTCH1, IL-1α, and IL-1β positive cells is significantly lower in the DAPT treated mice compared to control groups.

## 5. Conclusion

Regulation of proliferation, angiogenesis, and inflamation in CRC is related to an intact Notch, IL-1, and leptin signaling axis. Targeting Notch, IL-1, and leptin might help to design new pharmacological strategies aimed at controlling CRC growth and angiogenesis.
